# Improvised cardiac function of siddha formulation Sikkanjar elixir® through CAMK2 and VEGF mechanism-in experimentally induced cardiac hypertrophy model in rats

**DOI:** 10.1016/j.jaim.2026.101402

**Published:** 2026-10-12

**Authors:** S. Joseph Maria Adaikalam, J. Tejasri, R. Arunagiri, B. Naveen Kumar, M. Ramanathan

**Affiliations:** a Department of Research and Development, Earth India Naturals, Tiruvannamalai, TN, India; b Department of Pharmacology, PSG College of Pharmacy, Coimbatore, TN, India

**Keywords:** Cardiac hypertrophy, Sikkanjar elixir, Troponin 1, SIRT3, Endothelial function, VEGF

## Abstract

**Background:**

Sikkanjar elixir**®** (SE) contains 29 herbs, honey and fermented rice water. It is a modified and fortified form of *Sikkanjar Manappagu* (*Manappagu* means syrup), a Siddha traditional medicine. SE has its reference to treat vascular block, high cholesterol, varicose vein, hypertension; improved cardiac efficiency and blood circulation. However, the validation of Siddha claim of SE has not been carried out in experimentally conditions.

**Objective:**

The present study designed to validate the scientific claim of SE and investigate the cardio-protective prospective of SE, in a rat model of cardiac hypertrophy (CH).

**Materials and methods:**

CH was induced in Sprague-Dawley rats via ligation of the descending abdominal aorta. The cardio-protective effects of SE were evaluated through behavioral assessments, biochemical parameters, antioxidant profiling, liver and kidney function tests, ATP, troponin I levels measurement. The cardiac hypertrophy markers like CAMK2, VEGF, and SIRT3 expression were measured through qRTPCR.

**Results:**

HPTLC evaluation of SE denotes 1.221 mg RUE/g and 4.604 mg GAE/g, of total flavonoid and phenolics content respectively. CH induction led to elevated heart rate, troponin I, and liver/kidney function markers, along with decreased plasma glucose, cholesterol, tissue ATP, catalase, glutathione, and VEGF levels. SE treatment significantly reversed these changes, as evidenced by reduced heart rate, normalization of biochemical markers, and improved antioxidant status. Notably, SE treatment suppressed CAMK2 expression and upregulated VEGF, suggesting enhanced myocardial contractility. Histopathological analysis further supported these findings, showing reduced myocyte fibre thickness and septal volume.

**Conclusion:**

SE reversed the CH induced pathology through restoration of cardiac pumping efficiency (ejection fraction) via CAMK2 and VEGF mechanism. Normalization of cardiac output might contribute to the recovery of kidney, metabolic functions and promotion of anti-oxidant effect. These observations can also be extrapolated in CCF or MI conditions in clinical situations.

## Graphical abstract

Cardioprotective activity of Sikkanjar elixir® in abdominal aorta induced cardiac hypertrophy model in rats. SE reversed the cardiac hypertrophy induced changes through restoration of cardiac pumping through recovery of kidney, liver, metabolic functions, ATP level, promoting anti-oxidants, CAMK2 and VEGF mechanism.

**Figure:**
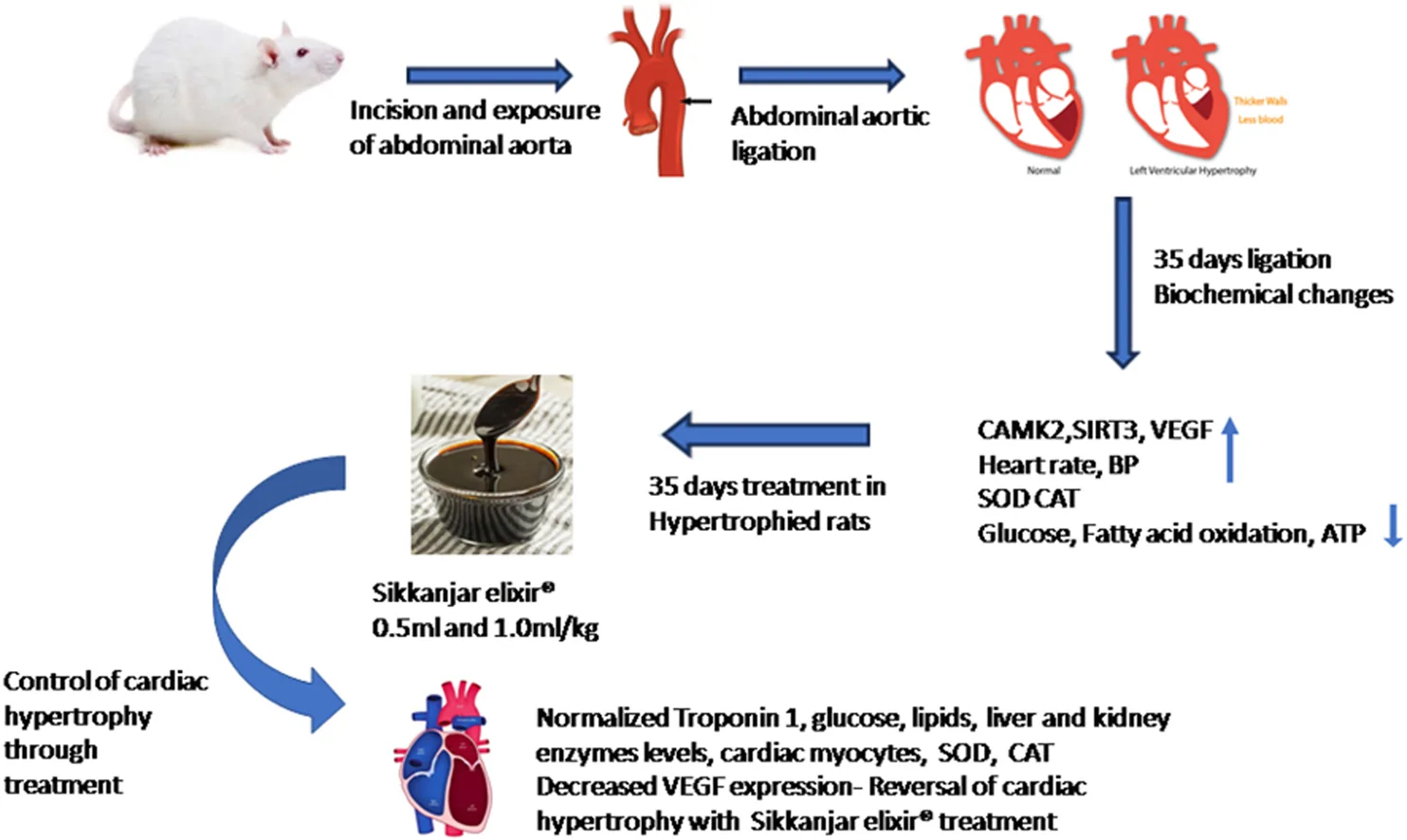

## 1 Introduction

Sikkanjar elixir**®** (SE) is a polyherbal syrup formulation – each herb added in SE has its reference in classical and approved textbooks of ancient literature of Siddha Medicine to treat vascular block, high cholesterol, varicose vein, hypertension, improve efficiency of heart and blood vessels. SE is a modified and fortified form of *Sikkanjar Manappagu* (*Manappagu* means syrup), a Siddha traditional medicine being in clinical practice for many decades. The SE contains 29 herbs, honey and fermented rice water (Table S1). The major ingredients of SE are *Allium sativum, Zingiber officinale, Terminalia arjuna, citrus fruits, Nelumbo nucifera, Sphaeranthus indicus, Mentha spicata Cissampelos pareira, Oxalis corniculata, Barringtonia acutangula, Bellis perennis, Cuminum cyminum Linn., Desmodium gangeticum, Tinospora cordifolia, Terminalia chebula, Emblica officinalis, Piper longum, Sida rhombifolia, Asparagus racemosus, Nervilia aragoana, Nelumbo nucifera*. These herbs have shown cardio protective, antioxidants, *anti*-hyperlipidemic and anti-inflammatory activities [[1], [2], [3], [4], [5], [6], [7], [8], [9], [10], [11], [12], [13], [14]] in experimental models. In the present study scientific validation of Siddha claim of SE was performed in experimentally induced cardiac hypertrophic conditions.

Neurohormonal factors, particularly angiotensin II, significantly contribute to the development of cardiac hypertrophy (CH). This occurs through a multi-faceted process. Angiotensin II (Ang II) stimulates the release of aldosterone, a hormone promoting sodium reabsorption and potentially contributing to increased workload on the heart. Further Ang II activates signalling pathways within myocyte that promote growth, leading to an increase in cell size. Additionally, Ang II can induce oxidative stress and inflammation, resulting in hypertrophic response [15]. Report showed SIRT-3 expression increased during early stages of experimentally induced CH model. This suggests SIRT-3 might be part of an initial compensatory response of the myocytes to adapt to increased workload [16]. However, SIRT-3 expression found to decrease as hypertrophy progresses to heart failure. This implies SIRT-3 function may be overwhelmed or become insufficient in later stages of the disease [17]. Studies have shown that inhibiting calcium/calmodulin dependent kinase (CAMK2) can prevent hypertrophy in animal models. Therefore, CAMK2 is more like a key player in the development of CH, rather than a simple marker [18]. Studies suggest that vascular endothelial growth factor (VEGF) is up regulated (increased) in response to CH, particularly in the early stages. This up regulation favours angiogenesis in the hypertrophic heart to maintain proper oxygen and nutrients supply [19,20]. The different pathways in which herbs mediate cardiac protective activity in myocardial injury have been reviewed [21,22].

The proposed study is intended to ascertain the Siddha formulary claim of cardio protective effect of SE through reverse pharmacological approach. The present work has been designed to figure out the cardio protective mechanism of SE in experimentally induced CH model. The observations are supported with general behavioral assessment, biochemical changes, antioxidant measurements, and hypertrophy molecular markers measurement through qRTPCR.

## 2 Materials and methods

**Chemicals and reagents**- Silk sutures were obtained from Healthium Medtech Pvt. Ltd. Povidone iodine (5%) was purchased from Cipla. Buprenorphine (0.05 mg/kg, IP) was procured from Neon Laboratories. An electrolyte solution consisting of sodium chloride, potassium chloride, sodium citrate, and dextrose was freshly prepared before use. Ketamine was obtained from Ciron Drugs and Pharmaceuticals Pvt. Ltd., while xylazine was sourced from Indian Immunologicals Ltd. Phosphate-buffered saline (PBS; composed of sodium chloride, potassium chloride, monobasic sodium phosphate, and dibasic sodium phosphate), nitro blue tetrazolium, bovine serum albumin (BSA), and Triton X-100 were all purchased from HiMedia Laboratories. Paraformaldehyde (4%), Tri reagent, and DTNB (Ellman's reagent) were obtained from Sigma-Aldrich and Thermo Fisher Scientific, respectively. Tris-HCl buffer was sourced from Sisco Research Laboratories Pvt. Ltd. Clinical biochemistry reagents including glucose, cholesterol, triglycerides, SGOT, SGPT, alkaline phosphatase, urea, uric acid, and creatinine were acquired from Agappe Diagnostics Ltd. Hydrogen peroxide was sourced from Nice Chemicals. Troponin I (rat cTn-I/TNNI3) assay kits were procured from Fine Test. ATP assay reagents were obtained from Elab science (Catalog No. *E*-BC-F002). A300 semi-autoanalyzer (Vital Scientific, MERCK) and a UV-Visible spectrophotometer (Shimadzu UV-1650–1700) were used for spectrometric measurements. cDNA synthesis was performed using a conversion kit from Thermo Fisher Scientific. Histological analysis was conducted using an H&E staining kit obtained from BioLab Diagnostics. Telmisartan (Telma 20) and Atenolol (Aten 25) were procured from Glenmark Pharmaceuticals and Zydus Cadila, respectively. The Siddha formulation SE**®** (Batch no PMAA 2502) was given as gift samples by the Earth India Naturals, Thiruvannamali.

**Preparation of SE-** The polyherbal Sikkanjar elixir**®** is a honey based liquid preparation, a licensed product manufactured and distributed among Siddha doctors and pharmacies since 2020 b y Earth India Naturals, Thiruvannamalai, a GMP certified company, under approval and license from the State Licensing Authority (Indian Medicine) of the Government of Tamil Nadu, India. The preparation contains 32 herbs. The indication of 32 herbs with its Siddha formulary text references is given in supplementary file Table S1. The detailed SE preparation is given in the supplementary file. In brief, SE is traditionally prepared with rice vinegar (*Kaadi*) and herbal/plant decoctions that are being used traditionally in Indian system of Siddha medicine since many centuries. First *Kaadi* is prepared by soaking cooked rice in water for one month, then straining the rice and allowing the water to ferment (Rice vinegar). The quality-checked herbal raw materials are weighed according to the proportions stated in the formulary. Coarse powder of the 12 barks and raisins of the formulation is soaked in the *Kaadi* to ferment for further one month. The decoction prepared with the remaining herbs is added to the fermented *Kaadi* and maintained at a medium temperature of 60°C −70°C to facilitate the extraction and blending of active constituents. The final liquid is filtered into a suitable vessel. Honey 40%w/v is added to the strained liquid and stirred until it dissolves. To remove any solid particles, SE is strained through a cheesecloth or fine mesh strainer. The final product syrup is allowed to cool and used clinically.

### 2.1 HPTLC studies

HPTLC analysis was conducted to identify compounds in the SE using CAMAG instrumentation. Methanol-predeveloped TLC aluminium plates (10 × 20 cm, silica gel 60F_254_ with fluorescence indicator) were used. Sample (0.1 μl) and reference standards (caffeic acid and p-coumaric acid; 0.4, 0.8, 1.2, and 1.6 μg) were applied as 9 tracks per plate using a CAMAG Linomat V applicator (dosage speed: 100 nL/s; first application position: x = 14 mm). Ascending development occurred in a twin-trough chamber (20 × 20 cm) pre-saturated for 30 min at 25°C ± 1°C and 50% ± 3% relative humidity, using the mobile phase Toluene:Ethyl acetate:Formic acid:Methanol (12:9:4:0.5, v/v/v/v) over a migration distance of 8.5 cm. Developed bands were visualized and photo documented under UV light (254 nm and 366 nm) in a CAMAG UV cabinet. Quantitative analysis was performed by scanning plates at 254 nm in absorption mode using a deuterium-tungsten (D_2_/W) lamp and evaluating chromatograms with winCATS 3.0 software.

**Preparation of extracts and standards**- Sample preparation for HPTLC involved concentrating the SE via solvent evaporation. Methanol was used as the solvent, and concentration was performed on a water bath. The concentrated solution was subsequently filtered to obtain a clear methanolic fraction, which served as the sample for HPTLC analysis. For use as chromatographic standards, caffeic acid and p-coumaric acid were separately dissolved in methanol to yield stock solutions of 400 μg/ml.

### 2.2 Determination of total flavonoid and phenolic content

Total flavonoid content in SE was determined following aluminium trichloride method. The total flavonoid content was calculated as rutin equivalents (mgRUE/g) with standard curve plotted with rutin. The total phenols present in the SE were measured by Folin-Ciocalteu's phenol method. The total phenolic content in SE was expressed as mg of gallic acid equivalents (mgGAE/g) [23,24].

**Animals-** 42 (21 males and 21 females) SD rats weighing 200-250 g were obtained from central animal house facility of PSG Health campus. Rats were housed in colony polypropylene cages. They were housed at 25 ± 2**֯**C with 50-55% RH and a 12:12 h light/dark cycle was maintained. They were provided with free food access and water *ad libitum*. They were randomly divided into 6 different groups. To minimize bias and account for potential sex differences in cardiac hypertrophy responses, sex was balanced across groups through stratified randomization using GraphPad Prism random number generator, with sex as a blocking factor; this ensured equal distribution (3 males and 3 females per group; n = 6/group total) and was implemented post-acclimatization but pre-surgery, in accordance with the NIH policy on Sex as a Biological Variable (SABV) for rigorous preclinical design (Clayton & Collins, 2014). After surgery, the rats were placed in individual cages and post-operative care was maintained. Group 1 was served as sham operated control. In remaining five groups CH was induced through surgery. Group 2 is negative control and received placebo. The placebo treatment indicates administration of lukewarm water 1 ml/kg which is recommended clinically for the dilution of the SE to facilitate its administration. The remaining four groups considered as treatment group and received telmisartan (TEL 5 mg/kg), atenolol (ATE 2 mg/kg) and the Siddha formulation Sikkanjar elixir® (SE 0.5 ml 1.0 ml/kg). Animal experiments were performed as per ICMR guidelines; “Guide for the care and use of laboratory animals”. The study protocol had been approved by the Institutional Animal Ethics Committee vide number 11/IAEC/2023 dated 28.04.2023.

**Drug preparation and treatment-** The marketed drug Telma-20 (telmisartan 20 mg) and Aten-25 (atenolol 25 mg) were purchased from the market. The drugs were prepared as suspension with 0.3% CMC as per the required concentration. The SE was mixed with equal proportion of lukewarm water before administration. All the drugs were administered orally in the morning and evening hours. The study period is 70 days. The heart rate of the rats was measured to confirm the CH development. From 35th day the treatment was started until the completion of the study period.

**Cardiac hypertrophy development-** CH was induced through ligation of descending abdominal aorta in ketamine (60 mg/kg) and xylazine (10 mg/kg) anesthetized rats. The procedure in brief, a 2 cm incision was made along the midline of the abdomen with a scalpel. The abdominal organs were carefully lifted and kept aside to locate the abdominal aorta. Aorta was isolated from connective tissue, particularly above the left renal artery. Silk suture thread was passed underneath of the abdominal aorta between the origins of the right and left renal arteries. Loose double knot was put with the suture thread to leave a 3 mm diameter loop and the cannula (0.9 × 40 mm) was placed longitudinally inside the loop. The knot around the aorta was tightened; the cannula was removed immediately to achieve a 0.7 mm diameter constriction. The internal organs were replaced, skin was closed by suture. In sham operated controls no aorta knot is performed. To prevent infection, povidone was applied and buprenorphine (0.05 mg/kg IP) were administered for pain management after recovery from anesthesia. Oral intake of fluids was monitored and incase oral intake was insufficient electrolyte solution were given. The rats were closely monitored for signs of infection, bleeding, or respiratory distress during post operative care [25,26]. The CH surgery was carried with the trained technician and the mortality rate within 48 h was found to be 14%. All the animals survived after 48 h and the required animals were replaced to maintain N. The rats after the sham operation recovered, and no mortality was found in this group.

**General behavior and observation-** The rats were observed for general behavioral changes after treatments using IRWIN scale. As per the procedure scores of 1, 2, 3, or 4 was graded based on the level of behaviors observed as slight, moderate, extreme, or severe [27]. The other parameters studied include body weight, food intake, water intake and heart rate during the treatment period. The heart rate of a rat was measured on day 35 and 71 after surgery. The LE 5002 non-invasive blood pressure system for rodents (Harvard apparatus) was used for this procedure.

**Blood collection and heart isolation-**The blood was collected by Retro-Orbital method on day 35th and 71st. The plasma was immediately separated from blood. The heart was carefully excised on day 71 along the cardiac and vascular borders after euthanasia. The heart was removed gently, without grabbing or manipulating the tissue. Weight of the heart was measured immediately after isolation. Heart was washed with phosphate buffer saline (pH = 7.4) and fixed with 4% paraformaldehyde for histopathology study. For RT-PCR and antioxidant study heart was stored at −80°C until processing. Approximately 80 mg of the heart tissue of control and CH samples were homogenized with 1 ml of Tri Reagent (Sigma Aldrich) using mortar and pestle for RNA isolation. The samples were then centrifuged at 12,000 × g for 15min at 2-8°C. For antioxidants measurement heart were homogenized with Tris-HCL buffer in a ratio of 1:10 w/v then centrifuged at 12,000 × g for 15min at 2-8°C. The supernatants were collected and stored at on the −80°C till analysis.

**Biochemical analysis-** The metabolic alterations of the Siddha formulation during 35 days treatment were assessed through measuring the biochemical changes in the plasma of different treatment groups. The biochemical measured are glucose, cholesterol, and triglycerides, liver function test SGOT (serum glutamic-oxaloacetic transaminase), SGPT (serum glutamic-pyruvic transaminase), alkaline phosphatase and kidney function test urea, creatinine and uric acid tests. All the function tests were performed using microlab300. The assay kits were purchased from Agappe Diagnostic Ltd., Protein level in the sample was estimated by Lowry et al. method [28].

### 2.3 Molecular measurement

**Antioxidants-** The antioxidant enzymes Superoxide dismutase (SOD), reduced glutathione and catalase were estimated in the left ventricle of the different treatment groups. Flohe and co workers [29] method was used to measure SOD level. This method monitors the enzyme's ability to inhibit the reduction of Nitro blue tetrazolium (NBT). One unit of SOD activity was defined as the amount of enzyme required to achieve a 50% reduction in NBT reduction.

The catalase activity was measured following Aebi method [30]. Briefly, 0.1 ml of the homogenate and 1.9 ml of 50 mM phosphate buffer (pH 7.0) was added. To this mixture 1 ml of freshly prepared 30 mM hydrogen peroxide (H_2_O_2_) solution was added to initiate the reaction. The rate of H_2_O_2_ decomposition by catalase was monitored using a spectrophotometer. The change in absorbance at 240 nm was measured at 30s intervals for 2min. Catalase activity is then expressed in units per milligram of protein (U/mg protein).

The determination of reduced glutathione was made by DTNB method. A reaction mixture consists of 0.5 ml of DTNB and 0.1 ml of tissue homogenate and 3 ml of 0.2 M phosphate buffer, and the resulting yellow color was measured at 412 nm.The activity of reduced glutathione was expressed as nmols/mg protein [31].

### 2.4 Quantification of troponin I by ELISA

The troponin I (pg/ml total protein) level in the heart tissue was measured Rat c Tn-I/TNNI3- ELISA kit (Fine test) specific for rats. ATP content in the heart tissue was measured in microlab300 semi auto analyzer using Vital scientific kit (Merck).

### 2.5 RT-PCR studies

Gene analysis in the heart tissues were measured by converting RNA into cDNA as per manufacturer's protocol. The study primers were designed and synthesized from NCBI primer-BLAST (sigma Aldrich MO, USA). The forward and reverse primers for rat VEGF are 5′-TTCATGGATGTCTATCAGCG-3′ and 5′-GCTCATCTCTCCTATGTGCT-3′, CAMK2; 5′-ATAGAAGTTCAAGGCGACCAG- 3′ and 5′-CAGCAAGATGTAGAGGATGAC G-3′ and SIRT3; 5′-GCTGCCAGCAAGGTTCTTAC-3′ and 5′-CCTTTCCACACCCTGGACTA-3′ respectively. The relative gene expression study was performed in duplicate in step one plus real-time PCR system (Applied biosystem, Grand Island, NY, USA). A melt curve was generated for every reaction to the confined product. Gene expression was normalized with β-actin and determined by ΔΔCT relative quantification method using a 2^−ΔΔCT^ equation.

### 2.6 Hematoxylin and eosin staining

Histopathology of the heart tissue was done to measure the CH development. The paraffin fixed heart muscle was cut into 5 μm thick sections. The cut sections were stained in hematoxylin and eosin stains. The myocyte thickness, length and nuclei formation was measured under 45X to measure the CH development and drug effects [32].

### 2.7 Statistical analysis

The statistical analyses of the data were performed using Graph pad Prism v8.0.2. The data were expressed as mean ± SD. The data were analyzed using one-way ANOVA followed by post hoc analysis with Dunnett's multiple comparison test and the probability level at p < 0.05 was considered as statistical significance.

## 3 Results

### 3.1 HPTLC and quantification of phenolics

HPTLC analysis confirmed the presence of p-coumaric acid (Rf 0.51) and caffeic acid (Rf 0.46) in the methanolic extract of SE, with chromatographic peaks corresponding to authentic standards (Fig. S1). Quantification revealed caffeic acid exhibited a peak area of 6060.3 AU and maximum peak height of 369.3 AU, while p-coumaric acid showed a peak area of 1819.32 AU and maximum peak height of 87.41 AU (Fig. S2). Calibration against reference standards yielded concentrations of 3.312 μg caffeic acid and 0.171 μg p-coumaric acid per application spot in the extract. The total flavonoid and phenolic content (Fig. S3a and b) present in SE were determined as 1.221mgRUE/g and 4.604 mgGAE/g respectively.

### 3.2 General behavior

*IRWIN scale*-the data representing general behavior is given in Fig. 1. Ligation of the descending aorta and allowed it for 35 days without treatment resulted in the development of CH. The general behavior of the animal was evaluated using IRWIN scale. The animals were observed for first 1 h after the drug administration for any behavioral alterations. The general behaviors observed with the animals include physiological functions of excitation, stereotype and autonomic functions. In the initial 35 days (non-treatment period) most of the rats have shown increased reactivity on touching. Animals have shown jumping behavior after ATE and TEL administration. In stereotype behavior at high dose the animals have exhibited sniffing behavior and few animals have shown scratching behavior. SE treated rats showed increased locomotor activity and alertness behavior.

**Fig. 1:**
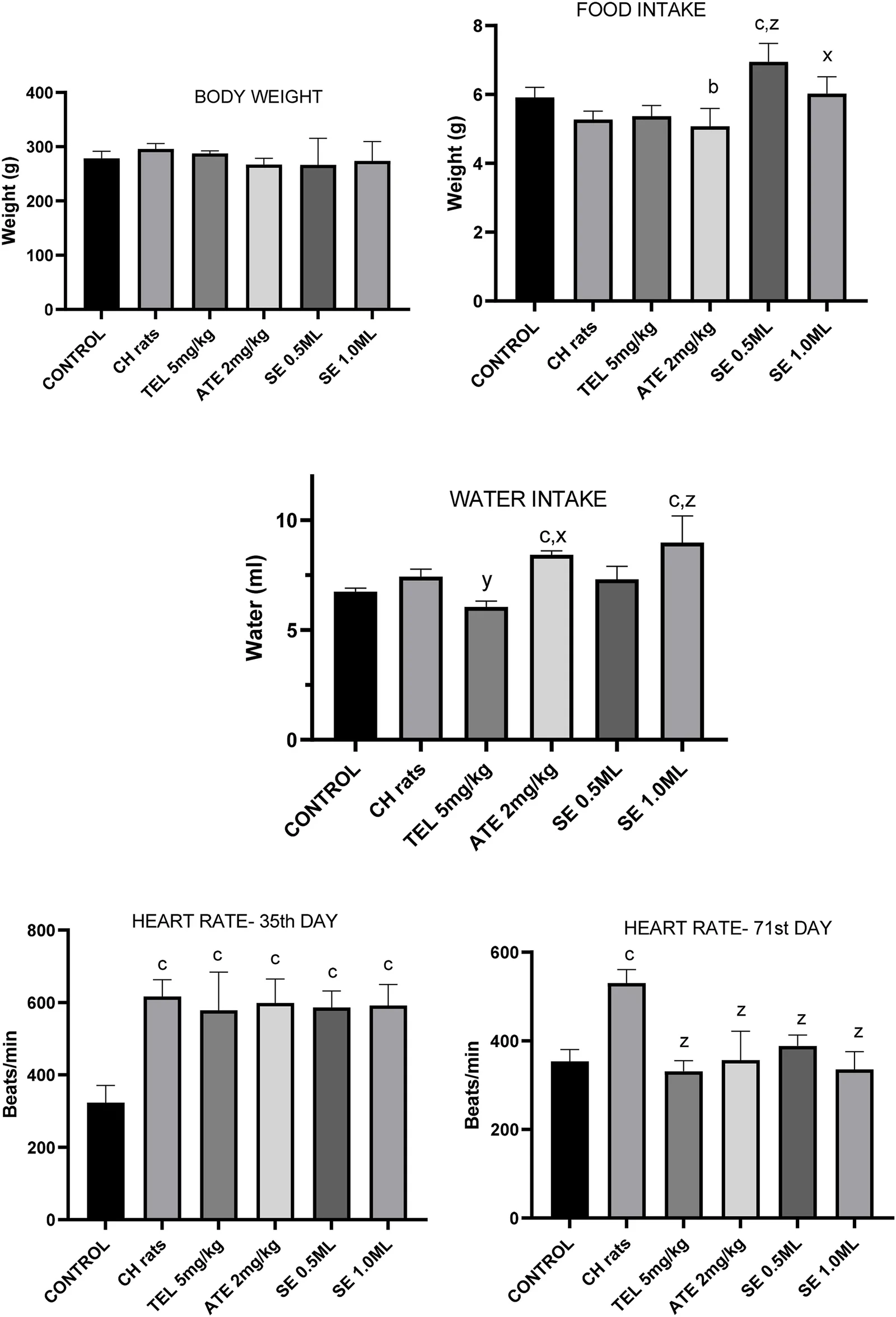
Effect of 35-day treatment with SE, initiated on the 35th day after surgery, on body weight, food intake, water intake, and heart rate (measured on days 35 and 71) in rats with cardiac hypertrophy (CH). Data are expressed as mean ± SD (N = 6). Superscripts b and c indicate statistical significance compared with control rats at p < 0.01 and p < 0.001, respectively. Superscripts x and z indicate statistical significance compared with placebo-treated CH rats at p < 0.05 and p < 0.001, respectively.

*Body weight and food intake*- The body weight of the CH rats and all the treatment groups did not differ (F_(5,30)_ = 1.365; ns) during the study period. The food intake (F_(5,30)_ = 19.18; p = 0.0001) and water intake (F_(5,30)_ = 23.58; p = 0.0001) was found to be altered with drug treatment in comparison to placebo treated CH rats. The food intake was found to be increased with SE (0.5 ml) and decreased with ATE treatment in comparison to placebo treated control rats. Similarly, significant increase in water intake was observed with ATE, (2 mg/kg) SE (1.0 ml) treated rats. A significant increase in food intake was observed with SE (0.5&1.0 ml) treated rats in comparison to placebo treated CH rats. A significant reduction in water intake was observed with TEL (5 mg/kg) treated rats. Whereas, ATE (2 mg/kg) and SE (1.0 ml) treated rats exhibited increased water consumption in comparison to placebo treated CH rats.

*Heart rate*- Ligation of descending abdominal aorta significantly elevated the heart rate in CH rats (F_(5,30)_ = 17.77; p = 0.001) in comparison to placebo treated control rats on 35^th^day after surgery. Treatment of TEL, ATE, SE (0.5&1.0 ml) for 35 days significantly decreased the heart rate on 71st day (F_(5,30)_ = 23.35; p = 0.0001) in comparison to placebo treated CH rats. The reduction in the heart rate with treatment group drugs & SE is comparable to control rats’ heart rate.

## 4 Biochemical analysis

### 4.1 Glucose, lipid profile and ATP

The CH developed rat plasma glucose (F_(5,30)_ = 49.47; p = 0.0001) and cholesterol (F_(5,30)_ = 17.21; p = 0.0001) levels were found to be significantly decreased in comparison to placebo treated control rats. Plasma triglycerides (F_(5,30)_ = 5.985; p = 0.001) and protein (F_(5,30)_ = 12.84; p = 0.0002) level significantly altered with different treatments in CH rats. Treatment of TEL (5 mg/kg), ATE (2 mg/kg), SE (0.5 ml) significantly increased the glucose level in comparison to placebo treated CH rates. Plasma cholesterol did not alter with the treatments. Plasma triglycerides level was significantly decreased with ATE (2 mg/kg) treatment in comparison to control as well as CH rats. Similarly, a slight significant decrease in protein level was observed with TEL (5 mg/kg) and SE (1.0 ml) treatment. All other treatments remained unaltered in this parameter. A significant decrease in ATP level was observed with CH rats in comparison to control rats. Treatment of TEL (5 mg/kg), ATE (2 mg/kg), SE (0.5 & 0.1 ml) significantly restored the depleted ATP level in cardiac tissue (F_(5,30)_ = 39.14; p = 0.0001). Treatment of Siddha formulation SE (0.1 ml) shown higher level of ATP than the control rats indicates over activation of cellular metabolic function (Fig. 2).

**Fig. 2:**
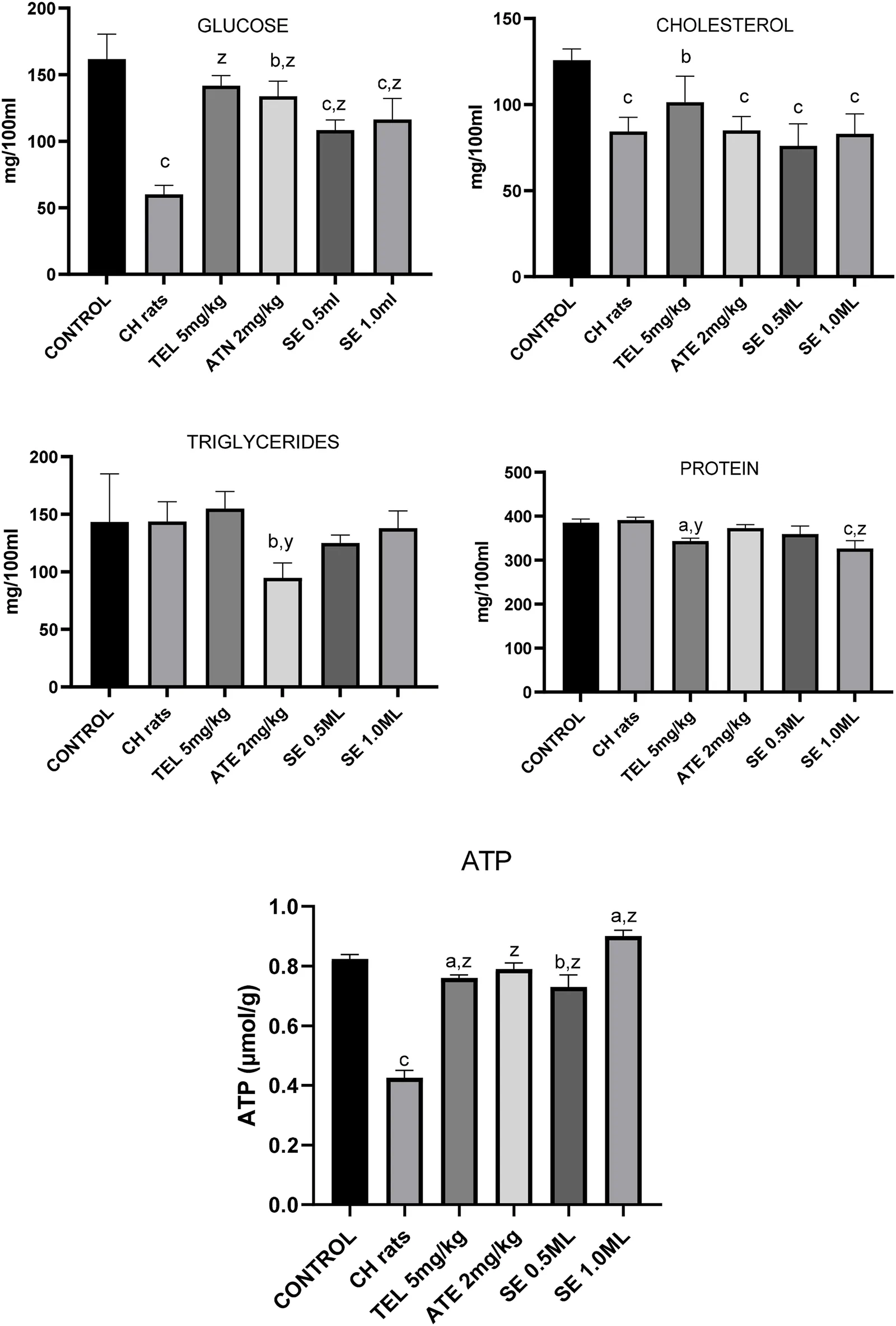
Effect of 35-day treatment with SE, initiated on the 35th day after surgery, on plasma glucose, cholesterol, triglycerides, protein and ATP levels in cardiac hypertrophy induced (CH) rats. Data are expressed as mean ± SD (N = 6). Superscripts a, b and c denotes statistical significance compared to control rats at p < 0.05, p < 0.01 and p < 0.001 respectively. Superscripts y and z denotes statistical significance compared with placebo treated CH rats at p < 0.01 and p < 0.00, respectively.

### 4.2 Liver function

The liver function indicative enzymes SGOT (F_(5,30)_ = 3.579; p = 0.01), SGPT (F_(5,30)_ = 7.397; p = 0.0001) and alkaline phosphatase (F_(5,30)_ = 6.64; p = 0.0003) level were found to be significantly elevated in placebo treated CH rats in comparison to placebo treated control rats. Thirty-five days treatment of SE (0.5& 1.0 ml) significantly decreased the elevated alkaline phosphatase level in comparison to placebo treated CH rats. SGOT level was found to be decreased with SE (1.0 ml) treatment. The elevated SGPT level was found to be significantly reduced with TEL, ATE, SE (0.5&1.0 ml) treatment groups in comparison to placebo treated CH rats (Fig. 3).

**Fig. 3:**
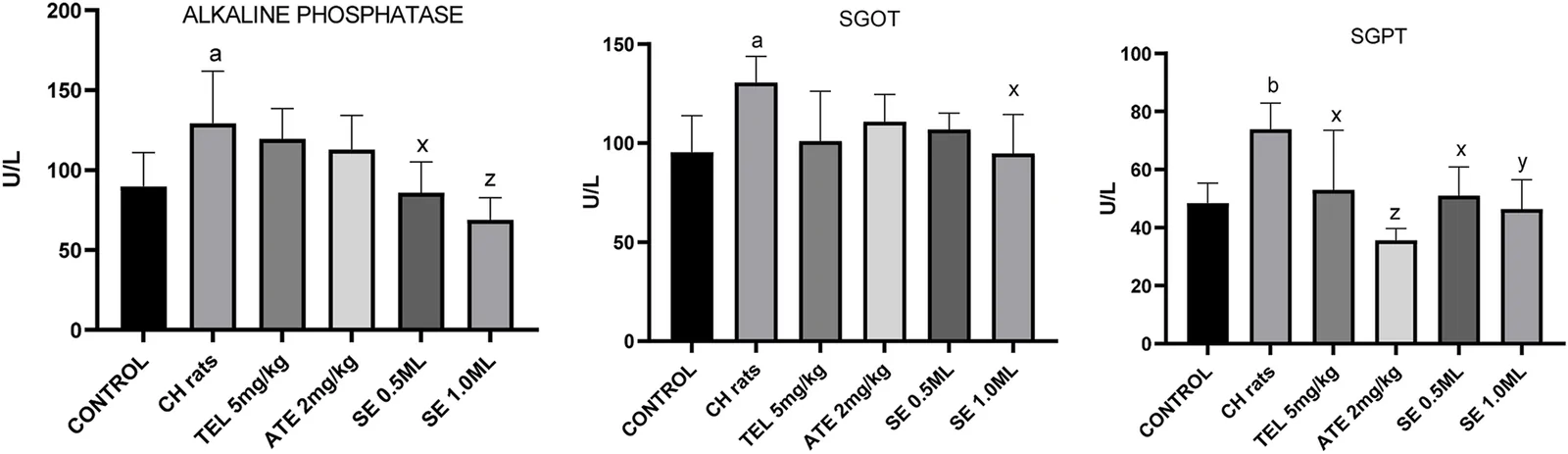
Effect of 35-day treatment with SE, initiated on the 35th day after surgery, on liver enzymes in cardiac hypertrophy (CH) induced rats. Data are expressed as mean ± SD (N = 6). Superscripts a and b denotes statistical significance compared with control rats at p < 0.05 and p < 0.01 respectively. Superscripts x, y and z denotes statistical significance compared with placebo treated CH rats at p < 0.05, p < 0.01 and p < 0.001 respectively.

### 4.3 Kidney function

A significantly elevated uric acid (F_(5,30)_ = 18.22; p = 0.0001) and creatinine (F_(5,30)_ = 52.27; p = 0.0001) level was observed with CH developed rats on day 71 in comparison to placebo treated control rats. Urea (F_(5,30)_ = 2.846; p = 0.03) level remains unaltered with CH rats. Treatment of TEL, SE (0.5& 1.0 ml) significantly decreased the elevated uric acid level in CH rats. Significantly decreased urea level was observed with SE (0.5 ml) in comparison to placebo treated CH rats. Other treatments did not alter the urea levels. All the treatments significantly decreased the elevated creatinine level. The treatment of SE (0.5 & 1.0 ml) normalized the creatinine level (Fig. 4).

**Fig. 4:**
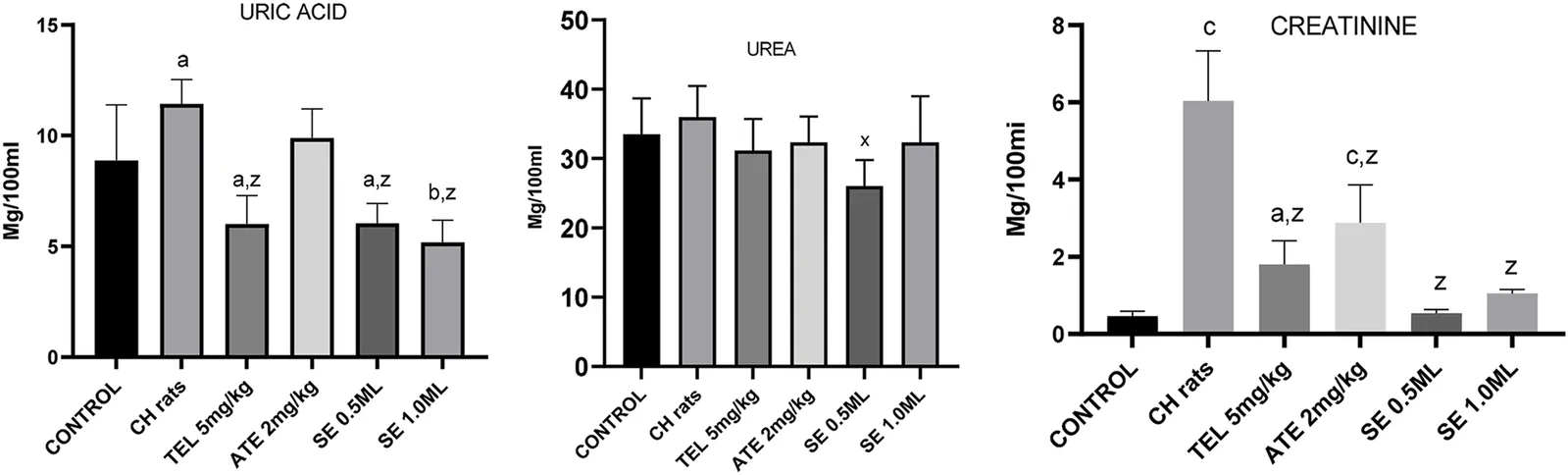
Effect of 35-day treatment with SE, initiated on the 35th day after surgery, on kidney function test in cardiac hypertrophy (CH) induced rats. Data are expressed as mean ± SD (N = 6). Superscripts a and c denotes statistical significance compared with control rats at p < 0.05 and p < 0.001 respectively. Superscripts x and z denotes statistical significance compared to placebo treated CH rats at p < 0.05 and p < 0.001 respectively.

## 5 Marker measurement

### 5.1 Antioxidant

Development of CH resulted in significant depletion of SOD (F_(5,12)_ = 25.95; p = 0.0001), catalase (F_(5,12)_ = 139.3; p = 0.0001), and glutathione (F_(5,12)_ = 16.85; p = 0.0001), levels in the cardiac muscle in comparison to placebo treated control rats (Fig. 5). Treatment of SE (0.5 and 1 ml) significantly increased the SOD level in comparison to both CH rats as well as control rats. TEL and ATE did not alter the SOD levels. Glutathione level was found to be significantly increased with ATE treatment in comparison to CH rats. All other treatments did not alter glutathione level. Treatment of TEL, ATE and SE (0.5 and 1 ml) significantly increased the catalase level.

**Fig. 5:**
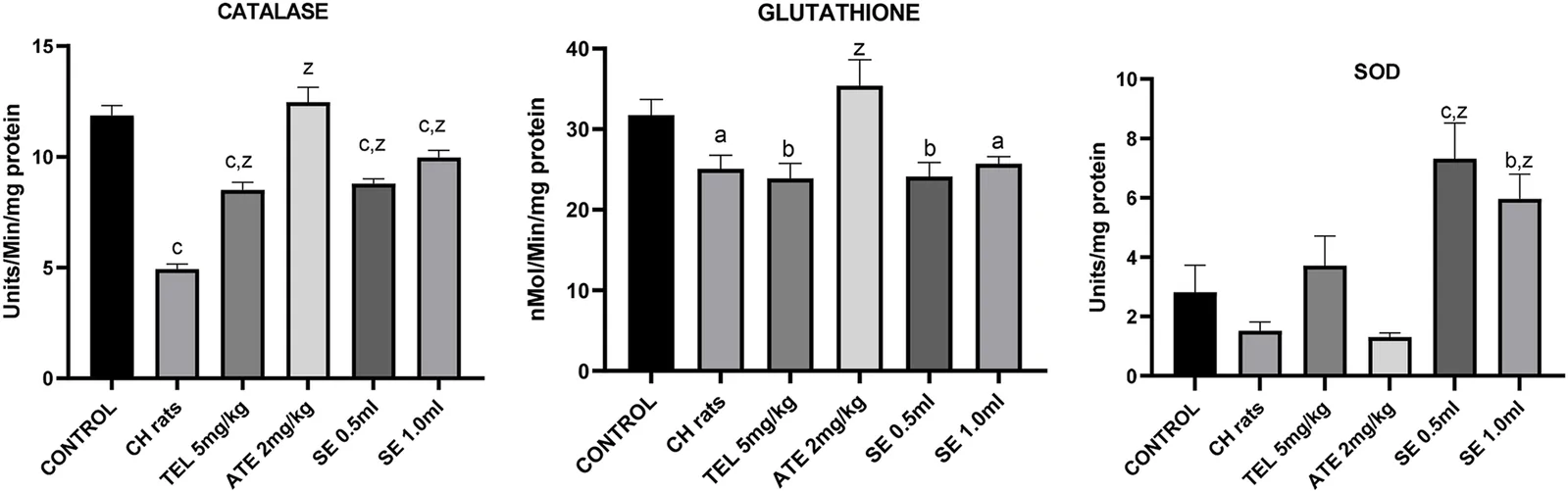
Effect of 35 days treatment of SE, initiated on the 35th day after surgery, on cardiac tissue catalase, glutathione and SOD levels in cardiac hypertrophy (CH) induced rats. Data are expressed as mean ± SD (N = 6). The superscripts a, b and c denotes statistical significance compared with control rats at p < 0.05, p < 0.01 and p < 0.001 respectively. Superscripts z denotes statistical significance compared with placebo treated CH rats at p < 0.001.

### 5.2 Troponin-I measurement

A significant increase in the serum troponin I level was observed in the placebo treated CH rats on day 35th (416.67pg/DL) as well as on 71st (563.52pg/DL) after the surgery in comparison to placebo treated control rats (363.61pg/DL). This clearly indicates that the cardiac tissue is under stress. Treatment of ATE 2 mg/kg (407.4pg/DL) and TEL 5 mg/kg (397.89pg/DL) significantly reduced the serum troponin I level in comparison to placebo treated CH rats. Treatment of SE (0.5 and 1.0 ml) significantly reduced the serum troponin I levels as observed by 329.67pg/DL and 352.17pg/DL respectively.

### 5.3 RT-PCR

Inducement of CH in rats significantly reduced the VEGF (F_(5,21)_ = 15.2; p = 0.0001) level in the cardiac tissue in comparison to placebo treated control rats. SIRT3 and CAMK2 level remains unaltered in CH rats (Fig. 6). Treatment of TEL, ATE and SE (0.5 & 1.0 ml) significantly increased the VEGF level. The reversal was complete and it was found to be equal to control rats’ VEGF level. SIRT3 level was found to be unaltered in CH rats (F_(5,21)_ = 1.643; ns) as well as with all the treatment groups. CAMK2 level (F_(5,30)_ = 7.922; p = 0.001) was found to be significantly reduced with TEL, ATE and SE (1.0 ml) treated rats in comparison to placebo treated control rats. Treatment of ATE (2 mg/kg) significantly decreased the CAMK2 level in comparison to placebo treated CH rats. Between SE (0.5 ml) and SE (1.0 ml) no significant difference was observed.

**Fig. 6:**
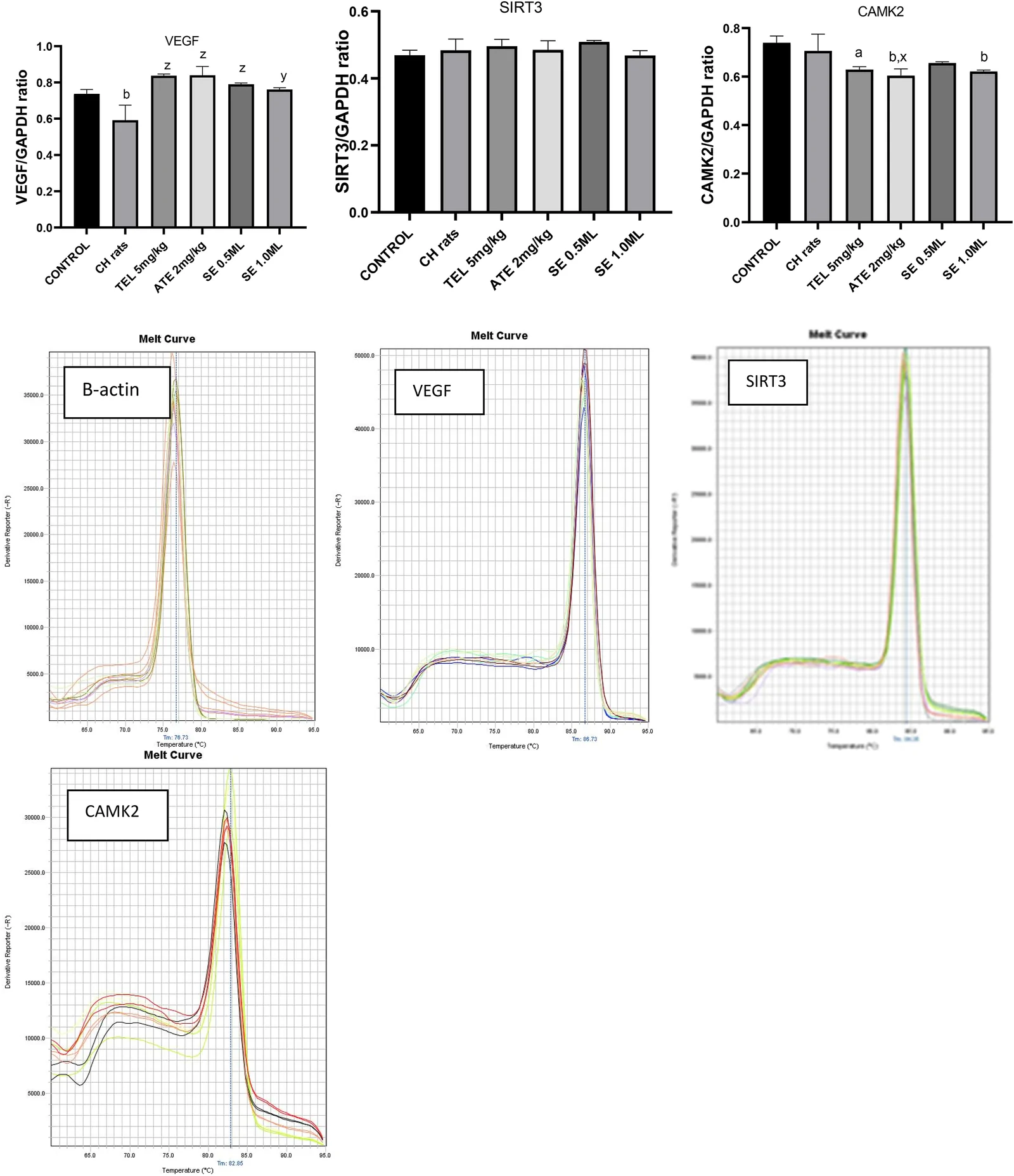
Effect of 35 days treatment with SE, initiated on 35th day after surgery, on VEGF, SIRT3 and CAMK2 gene expression in cardiac tissue in cardiac hypertrophy (CH) induced rats. Data are expressed as mean ± SD (N = 6). The superscripts a and b denotes statistical significance in compared with control rats at p < 0.05 and p < 0.01 respectively. Superscripts x, y and z denotes statistical significance compared with placebo treated CH rats at p < 0.05, p < 0.01 and p < 0.001 respectively.

### 5.4 Histopathology

The CH development was evaluated through measurement of heart weight, intraventricular septum measurement, left ventricular thickness measurement as well as the cardiac myocytes size. The excised heart and histopathology H & E staining of different treatment groups is given in Fig. 7. The placebo treated control rat's heart weight was found to be 3.41 mg/g body weight. CH rats' heart weight was found to be 3.87 mg/g body weight. CH rats treated with ATE and TEL have shown 3.46 and 3.52 mg/g body weight. Whereas CH rats received SE (0.5 and 1.0 ml) recorded 3.37 and 3.28 mg/g body weight. These observations clearly indicate treatment of drugs and SE significantly reduced the heart size resulting in the attenuation of cardiac hypertrophy development.

**Fig. 7:**
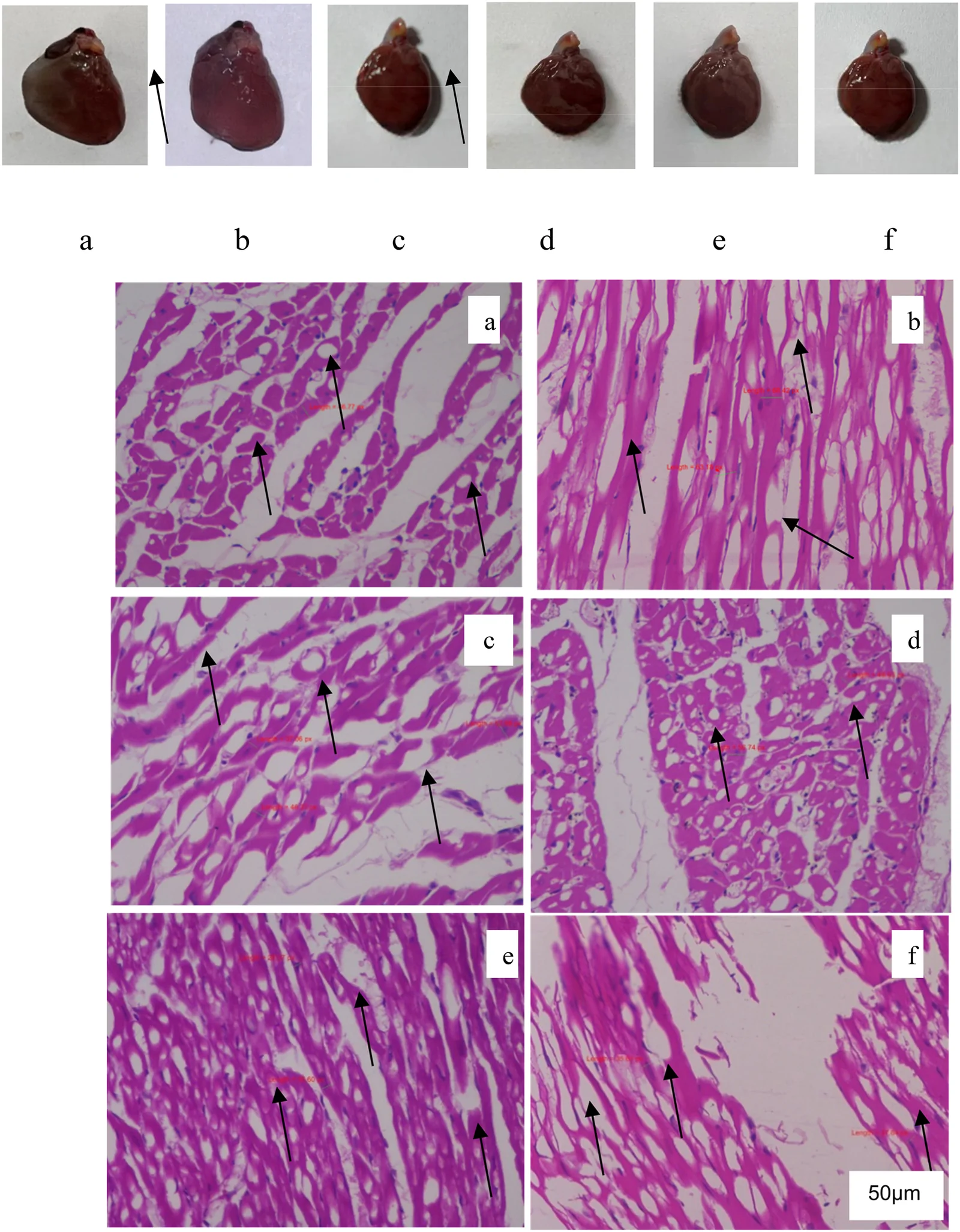
Representative H and E staining pictures of cardiac tissues showing myocyte morphology across experimental groups; a- Placebo treated control rats showing normal myocyte (size 46.77 μm^3^); b-placebo treated CH enlarged myocyte was observed (66.76 μm^3^) demonstrating development of cardiac hypertrophy c-atenolol (47.10 μm^3^) d-telmisartan (53.18 μm^3^); e and f - SE 0.5 ml (32.49 μm^3^); and 1 ml (36.83 μm^3^); shown decreased myocyte size in CH rats, Scale bar = 50 μm.

Hematoxylin and Eosin staining- Microscopical examination of the cardiac myocytes clearly denotes hypertrophy development in the cardiac muscle. In comparison to CH rat cardiac myocytes size (66.76 μm^3^), the cardiac myocytes of the placebo treated control rats were found to be significantly less (46.77 μm^3^). The SE treatment (0.5 & 1.0 ml) decreased the cardiac myocytes size 32.49 μm^3^ and 36.83 μm^3^ respectively. Per se beta blocker ATE (47.1 μm^3^) and angiotensin receptor blocker TEL (53.18 μm^3^) significantly decreased the cardiac myocytes size in comparison to CH rats.

## 6 Discussion

The model selected for the inducement of CH is one of the widely used experimental models in rats. Partial ligation of the descending abdominal aorta will develop resistance in the blood flow from the arch of aorta. This will indirectly increase the left ventricular contractile pressure in the heart to open the aortic valve. It will also increase the after-load of the cardiac muscle. Prolongation of this condition would result CH development leading to left ventricular dysfunction resulting in decreased stroke volume [25,26]. Further, to maintain cardiac output in CH condition as a compensatory mechanism the heart rate will increase. On 35th day after surgery the rats have exhibited increased heart rate and serum troponin I levels. The systolic pressure of the rats was also found to be elevated (data not shown). This observation confirms inducement of CH in rats. CH development was further confirmed with significant increase in the ventricular wall thickness along with intra ventricular septum volume. Biochemical markers VEGF, CAMK2 along with troponin I level in the cardiac tissue are the indicators of hypertrophy [17,18]. For the effective cardiac muscle contraction, the significant role of calcium ions along with sodium and potassium is essential. Renin angiotensin aldosterone system (RAAS) is considered on the prime regulator of blood pressure, ions and vascular functions [15]. In the present study it has been observed that the angiotensin receptor blocker TEL and β blocker ATE reversed the CH induced physiological and biochemical changes implies the role of adrenergic and RAAS in CH development [33,34]. Therefore, at this juncture we confirm the development of CH in rats after ligation of descending abdominal aorta. Clinically SE has been prescribed to patients reporting with chest pain associated with giddiness, chest discomfort and long-term cardiac illness. In the present study reversal of the CH induced pathological changes were observed with SE indicates cardioprotective effect of the Siddha formulation.

The herbs like garlic, ginger, citrus fruits and *T. arjuna* present in the SE are known to exhibit anti-inflammatory, anti-platelet aggregation, anti-oxidant activities and prevented cardiovascular diseases. The phytoconstituents present in these herbs are mainly flavonoids, flavones and glycosides [[1], [2], [3], [4], [5], [6], [7]]. Casuarinin phytoconstituent from *T. arjuna* inhibited phosphodiesterase 5 A enzyme and Na^+^K^+^ pump. Similarly, pelargonidin another phytochemical from *T. arjuna* inhibited phosphodiesterase 5 A and blocked β-adrenergic receptor activity [35]. *T. arjuna* exhibited cardioprotective through VEGF signalling pathway regulation and prevented CCF development [36]. Another herb in SE of *O. coniculata* aqueous extract exhibited cardioprotective activity in MI model [8]. The major constituent of SE is fermented rice bran, it acts like probiotic, regulates inflammation and gut microbiome. Earlier report indicated fermented rice bran with high probiotic content produced anti-inflammatory response in LPS treated macrophages [37]. Fermented black rice bran exhibited anti-obesity effects in 3T3-L1 pre-adipocyte cells and mice, suggests the beneficial probiotic effect of rice bran [38]. Therefore it can be stated that the combination of herbs in SE formulation would have produced synergistic cardio protective effect. Further, many studies have shown individual compounds effect in cardiac related pathology. The organosulfur compounds of garlic found to be interactive with MAPK, TGF-β, caspase-9, TNF-α and MMP-7, and targets in silico studies and qRTPCR studies have shown ameliorate action in glucose-induced cardiotoxicity [39]. Diallyl trisulfide compound from garlic has shown cardioprotective action in diabetic cardiomyopathy [40]. Phytoconstituent from ginger 6-gingerol improved the cardiac function and eases the transverse aortic re-constriction cardiac remodelling [41]. Zhang and co workers [42] have demonstrated attenuation of mechanical stress induced remodelling of cardiac tissue after transverse aortic constriction with astrangaloside IV targeting mitochondrial piezo1-VDAC1. These reports support our present findings.

Decreased glucose and cholesterol levels were observed in CH rats. Hypoglycemic status in the cardiac disease has been widely discussed by many researchers [43]. This might be a phenomenon due to the glucose utility or altered glycolysis. Earlier reports denote decreased ATP level in the cardiac disease which supports our present findings. Cardiac tissue mainly depends on ATP generated through fatty acid oxidation than glycolysis. Earlier reports indicate in the abdominal aorta constricted model of hypertrophy, fatty acid oxidation and its mRNA gene expression was found to be decreased with the slight elevation in glycolysis activity [44,45]. However, there is report stating that no change in the fatty acid oxidation in the experimental model [46]. Considering the biochemical changes (decreased lipid profile and increased glucose level) and ATP levels with SE treatment, in the present study, we state that the enhanced glycolysis mediated ATP molecule was produced to maintain the cardiac function to overcome the deficit fatty acid oxidation mediated ATP formation. Increased plasma SGOT, SGPT and alkaline phosphatase, creatinine and uric acid levels in CH rats might be attributed to the decreased blood flow or cardiac output distribution. The reason for the elevation of liver enzymes is not clear. Treatment of SE regulated the biochemical alterations without increasing the body weight. Depleted catalase, SOD and reduced glutathione along with ATP levels denote oxidative stress in the cardiac tissue and were reversed with SE treatment. The herbs of SE like *N. nucifera*, *C. cyminum*, *T. cordifolia*, garlic, *E. officinalis* and *T. chebula* are known for metabolic regulation, hypolipidemic activity and inhibition of HMGCoA enzymes [[9], [10], [11], [12], [13], [14]]. These herbs would have contributed the homeostasis.

SIRT3 was found to be a molecular marker in controlling the development of muscular hypertrophy by regulating oxidative stress in the muscular tissue and mitochondrial function. In the present study SIRT3 level was found to be unaltered with the treatments, the exact reason is unknown [17]. Recently it has been demonstrated that tanshinone IIA [47] an active compound from Traditional Chinese medicine, ginsenoside Rb1 [48], ligustrazine [49] phytoconstituents protected micro vascular endothelial cell injury through SIRT5 and associated signalling. In the current study we have observed similar effect with SIRT3 mechanism which supports our present results. The vasoendothelial growth factor-VEGF is responsible for angiogenesis. The role of VEGF has been discussed in the coronary heart diseases as well as in tumorigenesis. Increased expression of VEGF in CH rats indicates reduced vasculature growth. SE, TEL and ATE restored the VEGF growth level might be beneficial for the heart functioning. The CAMK2 is one of the biomarkers represents in the muscle contraction and found to be elevated in cardiac disease. In the CH muscle, the contractile power of the cardiac tissue will be less; hence measuring this kinase enzyme will indicate contractile efficiency with treatment. The molecule inhibiting the phosphorylation reaction of CAMK2 will prevent the downstream responses. In the present study, SE, TEL and ATE decreased the CAMK2 levels which indicate that these drugs might have improved the contractility of cardiac muscle through inhibition of phosphorylation [18]. Hence it can be stated that SE would have improved the cardiac pumping efficiency to restore the cardiac output in CH condition. This was further supported with histopathological data.

**Conclusion**- SE reversed the CH induced pathology through restoration of cardiac pumping efficiency (ejection fraction) via CAMK2 and VEGF mechanism. Normalization of cardiac output might contribute to the recovery of kidney, metabolic functions and promotion of anti-oxidant effect. These observations can also be extrapolated in CCF or MI conditions in clinical situations. Further studies, on other targets like TMBIM6-NDUFS4 can be explored as SE regulated glucose and lipid metabolism [50]. Cardiac protective activity measurement through cardio toxicity inducement [51], reperfusion injury models, and mitochondrial mechanism in ischemic or hypo-perfusion models can be studied to ascertain the cardiac mechanism of SE. The limitation of the study is pharmacokinetic parameters measurement.

## Author's contribution

SJMA is a Siddha doctor supported in formulation development, study design, data interpretation and manuscript preparation. JT helped in statistical analysis and manuscript preparation. RA helped in literature collection, biochemical analysis and manuscript preparation. BNK supported in study design, performed surgery, animal experimentation and statistical analysis. MR contributed in study design, conceptualization of ideas, manuscript preparation and data interpretation.

## Declaration of generative AI in scientific writing

Generative AI in scientific writing has not been used to write the manuscript and data interpretation.

## Funding

It is a collaborative work between Earth India Naturals and 10.13039/100005777PSG 10.13039/100006790College of Pharmacy. It is a self-funded research and no external funds received.

## **Declaration of competing interest**

The authors declare “They have nothing to declare".
