# Supplementary material for: Improvised cardiac function of siddha formulation Sikkanjar elixir® through CAMK2 and VEGF mechanism-in experimentally induced cardiac hypertrophy model in rats
Source: J Ayurveda Integr Med. 2026 Oct 12;17(5):101402. doi: 10.1016/j.jaim.2026.101402 (PMC13681581; doi:10.1016/j.jaim.2026.101402)
Supplement: Supplementary file 1 [file mmc1.docx]

**Supplementary file- Joseph et al.,**

**Title**- Improvised cardiac function of Siddha formulation Sikkanjar elixir^®^ through CAMK2 and VEGF mechanism- in experimentally induced cardiac hypertrophy model in rats

**Figure S1- HPTLC chromatogram of SE**

p-coumaric acid	Caffeic acid

HPTLC Chromatogram of SE

**Figure S2- AUC quantification of SE, p-coumaric acid and caffeic acid**

p-coumaric acid

Caffeic acid

**Figure S3- Quantification of total phenolics and flavonoids in SE**

Figure S3a- Determination of flavonoids

SE absorbance at 760nm is 0.592nm

Figure S3b- Determination of Total phenols

SE absorbance at 760mn is 0.880nm

**Table S1- Sikkanjar Elixir herbal composition**

| **S. No.** | **Scientific Name** | **Part Used** | **Quantity** | **Action*** | **Reference book** | **Page** |
| --- | --- | --- | --- | --- | --- | --- |
| 1. | Oriza sativa | Bran | 2ml | *Oothal, Peruvayiru* | SFI-I, Govt of India, 1st Ed, 1992 | 160 |
| 2. | Allium sativum | Root | 0.5g | *Nenjadaippu* | ARV, Thamarai Noolagam, 2000 | 605 |
| 3. | Zingiber officinale fresh | Root | 0.5g | *Rudravayu* | ARV, Jayam Printers, Madurai, 1st Ed | 487  488 |
| 4 | Citrus lemon | Fruit | 0.5g | *Rudravayu* | ARV, Jayam Printers, Madurai, 1st Ed | 487  488 |
| 5 | Terminalia arjuna | Bark | 0.5g | *Idhaya Noi* | SPI, Govt of India, 1st Ed, 1992 | 126, 128 |
| 6 | Mentha spicata | Leaf | 0.25g | *Marvali* | SRT-I, Rathna Naicker& Sons, 1991 | 167- 170 |
| 7 | Nelumbo nuceifera | Root | 0.25g | *Maradaippu, Irudhayasoolai* | SRT-II, Rathna Naicker, 1993 | 161 |
| 8 | Sphaeranthus indicus | Whole Plant | 0.25g | *Maradaippu, Irudhayasoolai* | SRT-II, Rathna Naicker, 1993 | 161 |
| 9 | Cissampelos pareira | Leaf | 0.25g | *Maradaippu, Irudhayasoolai* | SRT-II, Rathna Naicker, 1993 | 161 |
| 10 | Oxalis corniculata | Whole Plant | 0.25g | *Maradaippu, Irudhayasoolai* | SRT-II, Rathna Naicker, 1993 | 161 |
| 11 | Cissampelos pareira | Root | 0.25g | *Irudhayaadaippu, Mayakkam* | SRT, Rathna Naicker, 1991 | 123 |
| 12 | Barringtonia acutangula | Fruit | 0.25g | *Irudhayaadaippu, Mayakkam* | SRT, Rathna Naicker, 1991 | 123 |
| 13 | Bellis perennis | Flower | 0.25g | *Irudhayaadaippu, Mayakkam* | SRT, Rathna Naicker, 1991 | 123 |
| 14 | Cuminum cyminum | Dried Fruit | 0.125g | *Kirukiruppu* | SFI-I, Govt of India, 1st Ed, 1992 | 152 |
| 15 | Mesua nagassarium | Fruit | 0.125g | *Kuthuvayu* | SFI-I, Govt of India, 1st Ed, 1992 | 152 |
| 16 | Nelumbo nucifera | Flower | 0.125g | *Maarvali* | SRT, Rathna Naicker, 1991 | 119 |
| 17 | Desmodium gangeticum | Root | 0.125g | *Maarvali* | SRT, Rathna Naicker, 1991 | 119 |
| 18 | Nymphaea nouchali | Root | 0.125g | *Maarvali* | SRT, Rathna Naicker, 1991 | 119 |
| 19 | Tinospora cordifolia | Whole Plant | 0.125g | *Maarvali* | SRT, Rathna Naicker, 1991 | 119 |
| 20 | Nervia aragoana | Whole  Plant | 0.125g | *Maarvali* | SRT, Rathna Naicker, 1991 | 119 |
| 21 | Terminali chebula | Fruit | 0.125g | *Maarvali* | SRT, Rathna Naicker, 1991 | 119 |
| 22 | Terminalia bellerica | Fruit | 0.125g | *Maarvali* | SRT, Rathna Naicker, 1991 | 119 |
| 23 | Emblica officinale | Fruit | 0.125g | *Maarvali* | SRT, Rathna Naicker, 1991 | 119 |
| 24 | Zingiber officinale dried | Root | 0.125g | *Maarvali* | SRT, Rathna Naicker, 1991 | 119 |
| 25 | Piper nigrum | Fruit | 0.125g | *Maarvali* | SRT, Rathna Naicker, 1991 | 119 |
| 26 | Piper longum | Fruit | 0.125g | *Maarvali* | SRT, Rathna Naicker, 1991 | 119 |
| 27 | Diptheracanthus patulus | Whole  Plant | 0.0625g | *Maarvali* | SRT, Rathna Naicker, 1991 | 119 |
| 28 | Sida rhombifolia | Root | 0.0625g | *Maarvali* | SRT, Rathna Naicker, 1991 | 119 |
| 29 | Ipomea mauritiana | Leaf | 0.0625g | *Maarvali* | SRT, Rathna Naicker, 1991 | 119 |
| 30 | Asparagus racemosus | Root | 0.0625g | *Maarvali* | SRT, Rathna Naicker, 1991 | 119 |
| 31 | Tragia involucrate | Root | 0.0625g | *Maarvali* | SRT, Rathna Naicker, 1991 | 119 |
| 32 | Honey | - | 2g | *Rudravayu* | ARV, Jayam Printers, Madurai, 1st Ed | 487  488 |

SFI-I: Siddha Formulary of India, Volume – I, ARV-Athma Ratchamirtham Ennum Vaithiyasarasangiragam, SPI – Siddha Pharmacopoea of India, SRT-I & II – Sikitsa Rathna Deepam-Vol 1 & II

*English translation/equivalent to medical term

***Oothal****-*Anasarca, Generalized oedema	***Peruvayiru****-* Ascites	***Nenjadaippu****-* Gastric-Cardiac syndrome.

***Idhaya Noi****-* Heart disease	***Maradaippu****-* Myocardial infarction	***Kirukiruppu****-* Giddiness

***Mayakkam****-* Dizziness	***Maarvali****-* Chest pain, Angina pectoris

***Irudhayasoolai****-* Another term for chest pain with burning sensation. Localized and sustained pain in the cardiac area

***Irudhayaadaippu****-* A block probably in the blood circulation or hindrance to movement of Vayu (gas) in the chest area. May also refer to insufficient Cardio-respiratory functions

***Rudravayu****-* A condition with the symptoms of chest pain, breathlessness, with weakness or numbness of limbs, possibly due to Congestive cardiac failure. Here there is imbalance of ‘Vayu’ which is responsible for circulation/movement

***Kuthuvayu****-* Stabbing pain, particularly in the chest and abdomen areas of body
